# Is ‘Resilience’ Maladaptive? Towards an Accurate Lexicon for Climate Change Adaptation

**DOI:** 10.1007/s00267-015-0650-6

**Published:** 2015-12-31

**Authors:** Nicholas A. Fisichelli, Gregor W. Schuurman, Cat Hawkins Hoffman

**Affiliations:** Natural Resource Stewardship and Science, US National Park Service, Fort Collins, CO USA

**Keywords:** Conservation planning, Global change, Landscape conservation, Natural resources, Protected area management

## Abstract

Climate change adaptation is a rapidly evolving field in conservation biology and includes a range of strategies from resisting to actively directing change on the landscape. The term ‘climate change resilience,’ frequently used to characterize adaptation strategies, deserves closer scrutiny because it is ambiguous, often misunderstood, and difficult to apply consistently across disciplines and spatial and temporal scales to support conservation efforts. Current definitions of resilience encompass all aspects of adaptation from resisting and absorbing change to reorganizing and transforming in response to climate change. However, many stakeholders are unfamiliar with this spectrum of definitions and assume the more common meaning of returning to a previous state after a disturbance. Climate change, however, is unrelenting and intensifying, characterized by both directional shifts in baseline conditions and increasing variability in extreme events. This ongoing change means that scientific understanding and management responses must develop concurrently, iteratively, and collaboratively, in a science-management partnership. Divergent concepts of climate change resilience impede cross-jurisdictional adaptation efforts and complicate use of adaptive management frameworks. Climate change adaptation practitioners require clear terminology to articulate management strategies and the inherent tradeoffs involved in adaptation. Language that distinguishes among strategies that seek to resist change, accommodate change, and direct change (i.e., persistence, autonomous change, and directed change) is prerequisite to clear communication about climate change adaptation goals and management intentions in conservation areas.

## Introduction

“The beginning of wisdom is the definition of terms.”—Socrates

Concurrent with new challenges and scientific advances, ecological management concepts form and evolve, or go extinct. As our understanding of the natural world and our role within it deepens, conservation concepts, strategies, and the precision of language to describe these concepts also progress. Conscious, intentional management is particularly critical in the context of climate change, habitat fragmentation, pollution, nonnative species, and widespread extinction and extirpation, which compel conservation practitioners to operate at multiple scales and under unprecedented types and rates of change (Heller and Zavaleta 2009; National Park System Advisory Board 2012). As conservation science and management evolve to address these factors, examining conceptual terms that confuse rather than clarify climate change adaptation is warranted. The ubiquitous term ‘climate change resilience’ deserves scrutiny because: (1) its current use is ambiguous and often misunderstood, (2) it has different meanings across stakeholder groups and spatial scales, and (3) rapid directional change punctuated by amplified extremes compels candid disclosure of the likelihood of ecosystem shifts beyond historical ranges of variability. Climate change adaptation for conservation includes a range of strategies from resisting to actively directing change on the landscape and where the concept of resilience fits is no longer clear.

Climate change adaptation for conservation is a new endeavor for managers and many real and perceived implementation challenges exist such as management capacity, funding, stakeholder expectations, and science and technology needs. While laws such as the US Endangered Species and US Wilderness Act, as well as agency policies may constrain adaptation in some circumstances, analyses also indicate greater flexibility in laws and policies than many agency staff perceive (Joly and Fuller 2009; Jantarasami et al. 2010; Long and Biber 2014). We need to eliminate perceived hurdles and facilitate adaptation actions through clearly stated purposes, collaboration across jurisdictions, and communication with stakeholders, all of which require unambiguous concepts, goals, and strategies that are widely and consistently understood.

## A World of Change

**Fig. 1:**
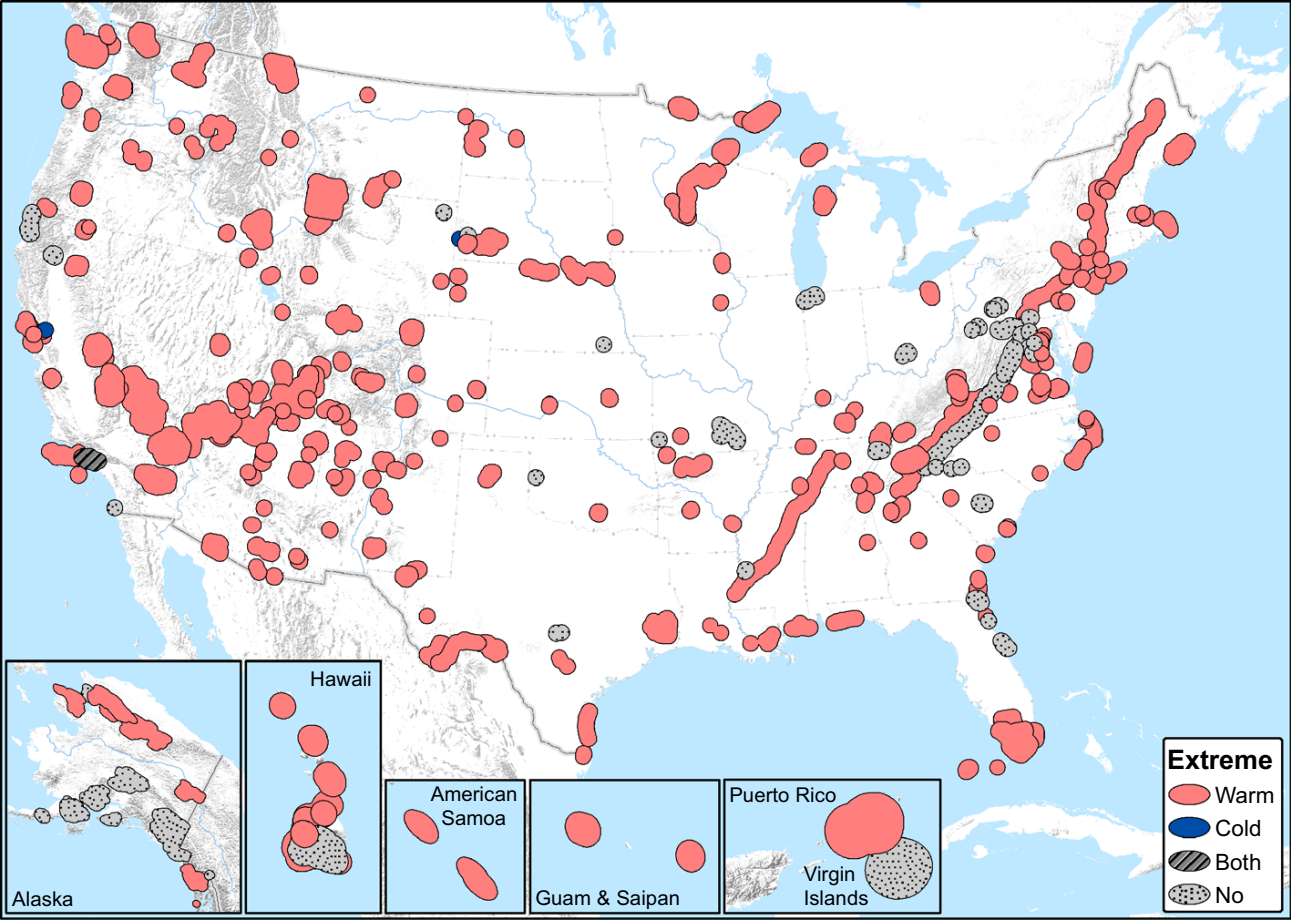
Recent (past 10–30 years) mean temperature relative to the historical range of variability (1901–2012) in 289 U.S. national parks (park plus surrounding landscape—30-km buffer). Park temperature is considered extreme if one or more of seven temperature variables examined is <5th percentile (‘Cold’) or >95th percentile (‘Warm’) of the historical distribution (adapted from Monahan and Fisichelli 2014)

The human footprint is ubiquitous on the planet such that many argue that we are now in the Anthropocene epoch (Steffen et al. 2007). Atmospheric CO_2_ is at its highest concentration in 800,000 years, driving increasing temperatures, sea level, and ocean acidity across the globe (IPCC 2013). Recent temperatures are already extreme relative to the long-term record—the past 30 years likely represent the warmest period in the northern hemisphere, on average, of the past 1400 years (IPCC 2013). Over 80 % of U.S. National Park System areas with significant natural resources (235 out of 289 parks) are already at the extreme warm edge of historical conditions (Fig. 1; Monahan and Fisichelli 2014). Beyond these recent changes, future combinations of temperature and precipitation in many areas may have no current analogs on the planet, making it difficult to predict potential ecosystem responses (Williams et al. 2007). Climate and other global change stressors not only challenge land managers’ abilities to protect and foster natural areas but also demand that we re-think conservation concepts, goals, and objectives in a continuously changing world (Hobbs et al. 2010).

## Ecological Processes and Change

Our understanding of ecological processes and the dynamic nature of ecosystems has expanded tremendously over the past 100+ years since the early work of Cowles, Clements, Gleason, and Tansley (Hagen 1992). For example, we no longer consider natural communities as superorganisms (Gleason 1926); we recognize ecosystems as dynamic and succession as typically non-linear (Pickett et al. 1987), and we acknowledge that “[climate] stationarity is dead” (Milly et al. 2008).

This last idea (Milly et al. 2008) deserves further thought. Climate is a fundamental driver of ecological processes and species distribution and abundance patterns on the landscape. Recent human-caused greenhouse gas emissions, long residence times of these gases in the atmosphere, and our current emissions trajectory suggest that the magnitude of future climate change will be substantial (Wigley 2005; Peters et al. 2013; Hansen et al. 2013) and thus ecological processes and species patterns will change significantly. Furthermore, anthropogenic climate change is not an episodic disturbance after which conditions return to a previous state; it is a combination of directional shifts in baseline conditions (e.g., increasing mean temperatures) and changes in extreme events (e.g., more frequent and intense storms and droughts). Viewed from a paleoecological perspective, the North American landscape has changed continuously with changes in climate (Davis 1983). Just as species responded individually to these changes with differing migration rates and pathways across the continent in the past (Davis 1983; Williams et al. 2004), current suites of species will disassemble and new, transient community types will form (Hobbs et al. 2009, 2010). Thus, in conservation areas—many of which were initially established to protect the biodiversity patterns present on the landscape (Groves et al. 2002)—preserving historical patterns of structure, composition, and location of natural communities over the coming decades and centuries is an unrealistic expectation.

## Climate Change Adaptation

Climate change adaptation is a new and rapidly evolving arena in conservation and management. Adaptation is an adjustment to actual or expected climate change and its effects that moderates harm or exploits beneficial opportunities (IPCC 2014). Numerous adaptation strategies, decision frameworks, and methods exist to facilitate climate change adaptation (West et al. 2009; NFWPCAP 2012; Stein et al. 2014), and adaptation practitioners often refer to adaptation strategies in three broad categories: resistance, resilience, and transformation (Millar et al. 2007). Resistance strategies seek to prevent climate change impacts to high-value and irreplaceable resources, whereas transformation strategies guide resource responses towards desired new conditions. Borrowing from the ecological definition of resilience [the amount of disturbance a system can absorb without changing states (Holling 1973; Gunderson 2000)], resilience strategies for climate change adaptation were initially described as supporting system recovery, and were “best exercised in projects that are short-term, have high amenity or commodity values, or under ecosystem conditions that are relatively insensitive to climate change effects” (Millar et al. 2007).

## Resilience: Muddying the Waters of Adaptation

**Fig. 2:**
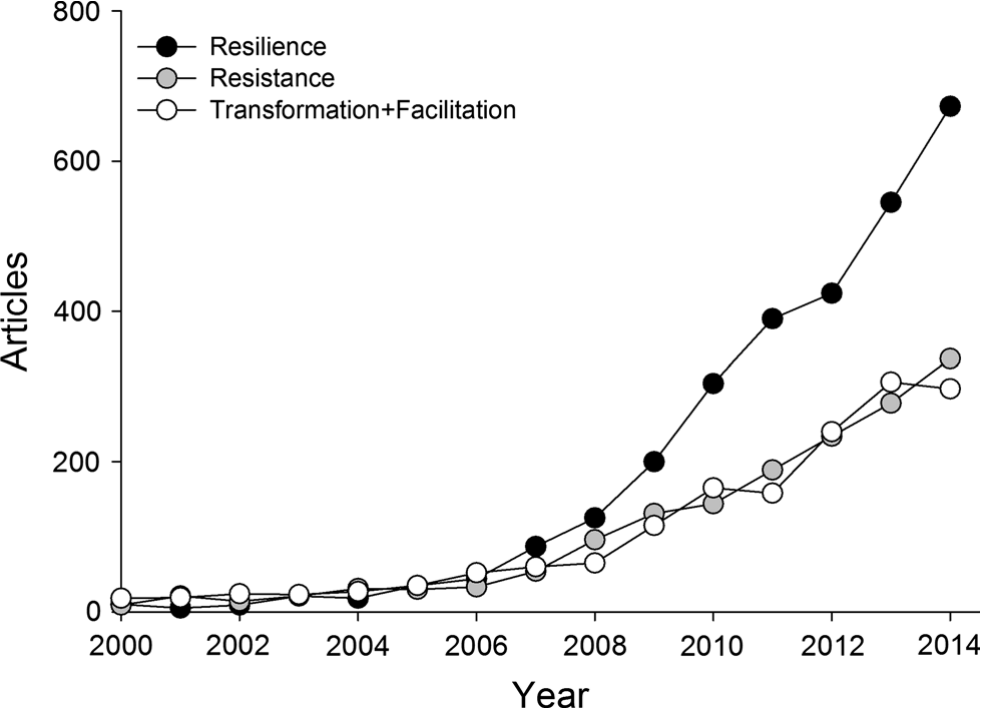
Number of English-language peer-reviewed scientific articles since 2000 in an academic citation index (Web of Science^®^ Science Citation Index Expanded) that contain both the words “climate change” and either “resilience,” “resistance,” or “transformation” or “facilitation”

‘Resilience,’ rather than gaining clarity with time, is increasingly confusing and ambiguous. Even before its use in the climate change lexicon, resilience as an ecological concept was becoming confusing due to multiple interpretations (Walker et al. 2004). Despite initial understanding within the conservation community of climate change resilience as a limited stopgap strategy, its use and definition in conservation and policy has expanded widely (Fig. 2), thus according “resilience” a very elastic quality, from “the amount of disturbance a system can absorb without changing states” (Holling 1973) to “the ability to anticipate, prepare for, and adapt to changing conditions and withstand, respond to, and recover rapidly from disruptions,” (Exec. Order No. 13653). Departing from the early adaptation concept of resilience (Millar et al. 2007) and borrowing from the social-ecological systems realm (Folke 2006), resilience now includes the full spectrum of climate change adaptation strategies, and encompasses the ability to *resist* change, or absorb change, or *transform* through response and self-organization (Carpenter et al. 2001; Chapin et al. 2010; Bernhardt and Leslie 2013; IPCC 2014).

Resilience is a positive, reassuring word implying strength, perseverance, and ultimate triumph over hardship, and any action may now claim to be one of ‘resilience’ in the name of adaptation. But is this catch-all label useful or is it a maladaptive term that confuses and impedes progress in climate change adaptation? Efforts to understand and address climate change impacts often bring together diverse assemblages of people from different backgrounds, sectors, and professional contexts (e.g., academic and agency scientists, engineers, adaptation specialists, land managers, policy makers, and public stakeholder groups), with attendant communication and linguistic challenges. Additionally, existing efforts illustrate that in response to the accelerating rate of climate change and its impacts, climate change adaptation best proceeds iteratively and collaboratively through science-management partnerships (Dilling and Lemos 2011; Halofsky et al. 2011). For example, a local-scale adaptation effort underway in the northern Great Plains includes actors from six federal agencies, two state-level programs, one academic institution, two tribal partners, and two non-profit organizations. Thus, the need to collaborate on climate change adaptation strategies at large spatial scales across management jurisdictions demands clear, commonly understood terminology, yet this elastic definition of resilience now renders the term as broad, vague, and uninterpretable as ‘naturalness’ (Yung et al. 2010). Decision frameworks for adaptation require defining conservation features, establishing clear management objectives, identifying necessary management actions, evaluating effectiveness, and revisiting and revising each step (Cross et al. 2012; Stein et al. 2014). The now-conflicting components of this expanded concept of resilience mean that objectives are simultaneously met and missed, depending on each stakeholder’s understanding of the term. At best, this expanded definition requires further specificity of sub-definitions to be useful in climate change adaptation (Morecroft et al. 2012).

The fact that the term resilience is common in everyday language further encourages divergent interpretations by, and thus confusion among, key stakeholder groups involved in climate adaptation, including administrative planners, conservation area managers, and the general public. Language matters, and popular understanding of the word resilience is “the ability of something to *return to its original shape* after it has been pulled, stretched, pressed, bent, etc.” (Merriam-Webster.com 2014). For natural resources and land management, this understanding refers to *existing* species, community types, and ecological processes. To many, a resilience strategy is a way to maintain historical fidelity and preserve current ecosystem states and processes in the face of perturbation (Higgs 2003; Cole et al. 2010). Thus, expectations of a resilience strategy may vary from resisting change for administrative planners and the public, to fostering directional change for some adaptation practitioners. Both of these goals may be desirable, but using the same word for both outcomes will result in perceived failure for at least one group, potentially damaging (often-tenuous) collaborative relationships.

Ongoing climate change further challenges the popular understanding of resilience by making it increasingly difficult to maintain both ecological patterns and processes on the landscape. Maintenance of ecological process in a changing world will often mean a change in patterns (e.g., community composition) on the landscape; conversely, maintaining current or historical species patterns may require intervention in ecological processes. For example, in areas where specific wildlife species depend on local vegetative biomass production (ecological process) for food, maintenance of adequate forage in the future may require a change in vegetation composition (pattern), whereas retention of historical plant species (adapted to past conditions) may cause a decline in biomass production and subsequent change in the wildlife community. As another example, wildfire activity and impacts in the western U.S. are already changing due in part to warming temperatures and drier conditions (Westerling et al. 2006; van Mantgem et al. 2013) such that a goal of maintaining current species assemblages may require additional management intervention aimed at altering the ecological process of fire. Maintaining pattern and process may be especially challenging in protected areas established to conserve features of scenery, species, communities, and ecological processes, thus heightening the need for transparency regarding goals and inherent tradeoffs in all discussions of climate change adaptation.

Transfer of a climate change resilience strategy from concept to implementation can also be difficult due to mismatches across disciplines and scales. Within social-ecological-systems, directed transformation of either or both the social and ecological aspects may be considered a resilience strategy. For example, the transformation of fish species assemblages found near a fishing village may require a transformation in the time, location, and equipment required to harvest newly abundant species in order to maintain a resilient fishery. Additionally, climate-mediated disturbance responses by human societies, such as behavioral changes and technological innovation, may occur over much shorter time scales than those required for ecosystem adjustment, such as the natural development of mature forests (Mace 2014). Thus, these differences in the time scale of responses further obscure what may be meant by resilience.

With respect to scale, natural resource management has generally been a species-centric undertaking, within relatively small management units (<1 million hectares) and over short time periods (years to decade) (Groves et al. 2002), whereas some recent definitions of resilience focus on ecological processes and functions at much larger (sub-continental) spatial scales and multi-decadal to centennial temporal scales (Zavaleta and Chapin 2010). While management focus on ecological processes and functions across larger landscapes is necessarily increasing, management at the species level still predominates and is required in many circumstances, such as for endangered and culturally significant species. We doubt many local managers or stakeholders would consider a strategy that enables current species within an area established in part for their protection to migrate out and a new suite of species to colonize the area as achieving resilience.

## Beyond Resilience

**Fig. 3:**
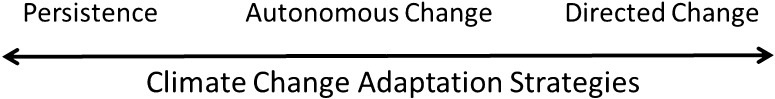
Climate change adaptation projects for conservation areas vary along a continuum of adaptation strategies. Appropriate options will vary over time, across space, and among resources

Based on ambiguity of the term, some climate change adaptation practitioners have already moved beyond the use of “climate change resilience” (e.g., Stein et al. 2014) and we similarly endorse a clearer more intuitive climate change adaptation lexicon for conservation areas that includes discernible management strategies (Fig. 3). This range of adaptation strategies includes ‘persistence’ of current conditions, ‘directed change’ towards a specific desired new future, and ‘autonomous change’ in which a resource responds to change with no management response intended to drive the system towards a specific state. Appropriate adaptation options will vary over time, across space, and among resources. The intensity of management intervention required to achieve goals depends on the focal resource’s vulnerability to climate change within the management area and may change with management time horizons and rates of climate change.

Inland fisheries management provides a useful illustration of this adaptation system. Under an ‘autonomous change’ strategy, managers would observe fish populations respond and self-organize as waters warm, and may manage other existing stressors (e.g., nonnative species) without intentionally directing the system towards a specific desired state. Some species will remain within the management area; others will migrate to more suitable locations or become extirpated, and yet other previously absent species that can reach the area will colonize this newly suitable habitat. In contrast, a goal to retain certain species would call for persistence strategies and trigger increased management intervention such as manipulating stream shading, reducing harvest, and/or increasing fish stocking levels. Under persistence strategies, as the climate continues to warm, management intensity needed to retain some historical species will increase and at some future point may exceed management capacity, or political will, and/or physical and ecological conditions may exceed species tolerances, at which point the strategy would no longer be viable. On the other end of the adaptation spectrum (‘directed change’), a goal of maintaining a recreational fishery regardless of species, for example, could prompt stocking of warm-adapted fish species (i.e., managed relocation). A major problem with the expanded resilience concept is that it now covers this entire climate adaptation spectrum, and thus is simply synonymous with the overarching concept of adaptation and therefore meaningless in communicating specific intent.

## Conclusions

Land managers and climate change adaptation practitioners currently confront both rapid directional change and multiple uncertainties (Heller and Zavaleta 2009). Ongoing climate change, other anthropogenic as well as natural disturbances, and our understanding of their interactions suggest that species and ecosystems will adapt and change, but are unlikely to return to pre-existing states because the underlying conditions no longer exist. Consistent with conservation practices, climate change adaptation begins with determining objectives, desired future conditions, and management strategies to achieve these goals. Successful adaptation typically is cooperative, cross-jurisdictional, and interdisciplinary. Viral proliferation and nebulous application of resilience is hindering adaptation practice and advancement. Adaptation requires clear and consistent terminology among collaborators and with stakeholders who deserve to understand the realities of climate change and consequences for species, resources, and landscapes. Climate change is ongoing; ecosystem change is inevitable, and successful climate change adaptation in conservation areas requires clear, direct language that distinguishes strategies that seek to resist change from those that direct change.
